# Lack of associations between two previously identified susceptible single nucleotide polymorphisms of interleukin-23 receptor gene and ankylosing spondylitis: a replication study in a Chinese Han population

**DOI:** 10.1186/1471-2474-14-190

**Published:** 2013-06-17

**Authors:** Bang-ping Qian, Jun Jiang, Ming-liang Ji, Bin Wang, Yang Yu, Yong Qiu

**Affiliations:** 1 Department of Spine Surgery, The Affiliated Drum Tower Hospital of Nanjing University Medical School, Nanjing, China

**Keywords:** Interleukin-23 receptor, Gene, Single nucleotide Polymorphism, Ankylosing spondylitis

## Abstract

**Background:**

The human leukocyte antigen (HLA)-B27 gene is considered to be a major gene associated with predisposition to ankylosing spondylitis (AS); however, studies have demonstrated that non-HLA-B27 genes also contribute substantially to the susceptibility to AS. Two single nucleotide polymorphisms (SNPs), rs1004819 and rs10889677, of the interleukin-23 receptor (IL-23R) gene have been shown to be associated with AS susceptibility in European populations. However, ethnicity factors contribute to population splitting and genetic variation, and ethnic-specific genetic association studies are needed to validate these associations in patients from different ethnic backgrounds. This study therefore aimed to replicate the associations between these two SNPs and AS susceptibility in a Chinese Han population.

**Methods:**

A total of 195 AS patients and 203 normal controls were recruited in this study. Two IL-23R gene SNPs, rs1004819 and rs10889677 were selected. Genotyping was performed in all subjects using the TaqMan probe method. Genotype and allele frequencies were compared between AS patients and normal controls by χ^2^ tests.

**Results:**

There were no significant differences in either the genotype frequencies (TT 36.4%, TC 48.7% and CC 14.9% in AS patients; TT 35.0%, TC 50.0% and CC 15.0% in normal controls) or allele frequencies (T 60.8% and C 39.2% in AS patients; T 60.0% and C 40.0% in normal controls) of rs1004819 between AS patients and normal controls (P > 0.05). In addition, both the genotype frequencies (AA 51.3%, AC 43.1% and CC 5.6% in AS patients; AA 57.6%, AC 35.5% and CC 6.9% in normal controls) and allele frequencies (A 72.8% and C 27.2% in AS patients; A 75.4% and C 24.6% in normal controls) of rs10889677 were also comparable between AS patients and normal controls (P > 0.05).

**Conclusions:**

This study found no evidence for an association between either of the two previously identified AS-susceptibility IL-23R SNPs (rs1004819 and rs10889677) and onset of AS, indicating a possible difference in pathogenesis of AS between Chinese and European patients.

## Background

Ankylosing spondylitis (AS) is an autoimmune-related disease characterized by chronic inflammations and gradual ossifications of the spine and sacroiliac joints [1]. Substantial evidence suggests that genetic predisposition has a significant impact on individual susceptibility to AS [2]. Although the human leukocyte antigen (HLA)-B27 gene is considered to be a primary gene associated with predisposition to AS and confers disease susceptibility, it is unable to explain all cases of AS and its contribution to the overall genetic predisposition is only 20–30% [3]. Increasing evidence suggests that other, non-HLA-B27 genes also play crucial roles in AS susceptibility [4-6].

The etiology of AS is characterized by aberrant regulations of both the adaptive and innate immune responses [7], suggesting that inflammatory mediators involved in the regulations of immunological reactions could be potential candidate genes. The interleukin-23 receptor (IL-23R) is a member of the hematopoietin receptor superfamily, and constitutes a specific component of the heterodimeric receptor for the cytokine IL-23 [8]. IL-23R plays a key role in the differentiation of CD4 T-cell into TH17 lymphocytes, which mediate inflammations in several animal models of autoimmunity [9,10]. IL-23R gene polymorphisms may thus be implicated in susceptibility to human autoimmune diseases, such as AS [11].

A recent genome-wide association study reported that the IL-23R gene conferred susceptibility to AS [12], while IL-23R gene variation was responsible for 9% of the population-attributable risks of AS [12]. The associations between IL-23R gene polymorphisms and AS susceptibility have been confirmed in European populations by genetic association studies [13-15], though the genotype distributions may differ among patients with different ethnic backgrounds [16]. Ethnic-specific genetic association studies are therefore needed to validate the associations between IL-23R gene polymorphisms and AS susceptibility. The aim of this study was to reevaluate the identified associations between two IL-23R gene single nucleotide polymorphisms (SNPs), rs1004819 and rs10889677, and AS susceptibility in a Chinese Han population.

## Methods

### Subjects

The study population consisted of 195 sequential unrelated AS patients (183 men and 12 women) and 203 age- and gender-matched normal controls (178 men and 25 women) recruited from January 2008 to January 2012. All the subjects investigated were of Chinese Han ethnicity. All the AS patients were recruited from the outpatient clinic of the affiliated Drum Tower Hospital of Nanjing University Medical School, while the normal controls were recruited from the medical control center for the healthy population at the hospital. The average age of the AS patients was 32 years (range, 21–53 years), and all patients satisfied the modified 1984 New York criteria [17]. The normal controls were healthy subjects with no autoimmune or musculoskeletal disease, with an average age of 37 years (range, 26–57 years). One hundred and ninety-two AS patients (98.5%) were HLA-B27-positive and all the normal controls were HLA-B27-negative.

Ethical approval was obtained from the research ethics committee of the medical school of Nanjing University and informed consent for DNA analysis was obtained from all subjects.

### Genotyping

Genomic DNA was extracted from peripheral blood leukocytes using genomic DNA isolation kits (Promega, Madison, WI, USA) according to the manufacturer’s instructions. Two IL-23R SNPs (rs1004819 and rs10889677) were selected for analysis. Genotyping was performed using the TaqMan Probe method with an ABI 7500 Real-time PCR system (Applied Biosystems, Foster City, CA, USA). The primers were designed by Primer Premier 5.0 and are listed in Table 1. The polymerase chain reaction system consisted of 5 μl 2 × TaqMan Master Mix, 0.5 μl of each primer (10 nmol/l), 0.25 μl of each probe (10 nmol/l), 2 μl DNA sample and 1.5 μl of sterile deionized water. The mixture was incubated for 5 min at 94°C, followed by 35 cycles of 94°C for 30 s, 58°C (for rs1004819) or 60°C (for rs10889677) for 30 s, 72°C for 1 min, and a final extension at 72°C for 7 min. Of these samples, 10% were tested twice to validate the genotyping results, with 100% reproducibility.

**Table 1:** Sequences of primers and probes for SNP rs1004819 and rs10889677

**SNP**	**Products**	**Sequence**
rs1004819	Top primer	5-CACTGACCTGCTTTATGCTGTGA-3
	Bottom primer	5-TTATAGCAGCACAAGCATTCTAGGA-3
	Probe1-FAM	FAM-TCTTACTATGCTATCTGC-MGB
	Probe2-HEX	HEX-TCTTACTGTGCTATCTG-MGB
rs10889677	Top primer	5-CGGGAGCTCCATGCCTTT-3
	Bottom primer	5-CACCTTCGGGACCTTAATTCTCTAA-3
	Probe1-FAM	FAM-TCTGCCTAATTTCT-MGB
	Probe2-HEX	HEX-CTTCTGCCTCATTTC-MGB

### Statistical analysis

Statistical analysis was performed using SPSS software (version 16.0, SAS, Inc, Chicago, IL, USA). The genotype and allelic frequencies in AS patients and normal controls were compared using χ2 tests. P < 0.05 was considered to be statistically significant.

## Results

The genotype frequencies of these two SNPs did not deviate significantly from the Hardy-Weinberg equilibrium in either the AS patients or controls.

The genotype frequencies for rs1004819 were TT 36.4%, TC 48.7% and CC 14.9% in AS patients, and TT 35.0%, TC 50.0% and CC 15.0% in normal controls. The allele frequencies were T 60.8% and C 39.2% in AS patients, and T 60.0% and C 40.0% in normal controls (Table 2). Neither the genotype nor the allele frequencies of rs1004819 differed significantly between AS patients and controls (P > 0.05). The genotype frequencies for rs10889677 were AA 51.3%, AC 43.1% and CC 5.6% in AS patients, and AA 57.6%, AC 35.5% and CC 6.9% in normal controls. The allele frequencies were A 72.8% and C 27.2% in AS patients, and A 75.4% and C 24.6% in normal controls (Table 2). The genotype and allele frequencies of rs10889677 were also comparable between AS patients and normal controls (P > 0.05).

**Table 2:** Allele and genotype frequencies of SNP rs1004819 and rs10889677 in ankylosing spondylitis patients and normal controls

**SNP**	**Genotype and Allele**	**AS patients N (%)**	**Normal controls N (%)**	**χ2**	**P**
rs1004819	TT	71 (36.4%)	71 (35.0%)	0.09	0.96
	TC	95 (48.7%)	101 (50.0%)		
	CC	29 (14.9%)	31 (15.0%)		
	T	237 (60.8%)	243 (60.0%)	0.07	0.79
	C	153 (39.2%)	163 (40.0%)		
rs10889677	AA	100 (51.3%)	117 (57.6%)	2.46	0.29
	AC	84 (43.1%)	72 (35.5%)		
	CC	11 (5.6%)	14 (6.9%)		
	A	284 (72.8%)	306 (75.4%)	0.67	0.41
	C	106 (27.2%)	100 (24.6%)		

## Discussion

A substantial proportion of AS genetic susceptibility is known to be encoded by non-HLA genes [5], among which the IL-23R gene has been reportedly associated with the onset of AS in European populations in previous genetic association studies [13-15]. The IL-23R SNPs rs1004819 and rs10889677 have both been found to confer risks of AS susceptibility in British [13], Hungarian [14] and Portuguese populations [15]; however, ethnic factors contribute greatly to population splitting and genetic variation and genotype distributions may differ significantly among different ethnic groups [16]. The SNP rs1004819 is an A/G variation in these European populations, but a C/T variation in the Chinese Han population. Additionally, although rs10889677 is an A/C variation in both European and Chinese Han populations, the allele frequency distribution of this SNP differs significantly between these populations, according to the results of previous European studies (Table 3). Data obtained from the International Hapmap Project (http://hapmap.ncbi.nlm.nih.gov/) also demonstrated wide variations in allele frequencies for these two SNPs between different races. The associations between these two SNPs and AS susceptibility demonstrated in European populations thus need to be confirmed in the Chinese Han population.

**Table 3:** Allele frequencies of SNP rs10889677 in European and Chinese Han populations

	**Ankylosing spondylitis patients (%)**				**Normal controls (%)**			
**Allele**	**A**	**C**	**Number**	P*	**A**	**C**	**Number**	P*
British [12]	36%	64%	1818	0.000	28.5%	71.5%	1331	0.000
Hungarian [13]	34.5%	65.5%	206	0.000	31%	69%	235	0.000
Portuguese [14]	28%	72%	358	0.000	36%	64%	285	0.000
Chinese Han	72.8%	27.2%	195		75.4%	24.6%	203	

*compared with Chinese Han population.

The IL-23R gene encodes an important cytokine receptor in the Th17 subset of T lymphocytes [18,19], and IL-23R, together with IL-12Rβ1, comprises the IL-23 receptor complex in IL-23-responsive cells [20]. IL-23 is one of the major regulators of innate and adaptive immunities [8]. It stimulates a unique CD4+ inflammatory T-cell characterized by secretion of IL-17, tumor necrosis factor and IL-6, which are strongly associated with proinflammatory responses and severe autoimmunity [8]. These conclusions suggest a possible involvement of the IL-23/IL-23R pathway in the etiology of AS. SNP rs1004819 and SNP rs10889677 are located in the intronic region and 3’-untranslated (3’-UTR) region of the IL-23R gene, respectively [7]. rs1004819 may exert its influence by regulating differential splicing of IL-23R mRNA, while rs10889677 may lead to variation in the 3’-UTR region, which could be associated with reduced binding of IL-23R mRNA [14]. Both these variations may have functional consequences for the IL-23R pathway and could provide a possible explanation for the associations between these two variants and AS susceptibility in European populations.

Despite the positive associations between these two SNPs and AS in European populations, neither rs1004819 nor rs10889677 was found to be related to AS susceptibility in the current study. The genotype and allele frequencies of both SNPs were similar in AS patients and controls, indicating that neither variation in the IL-23R gene was relevant to the etiology of AS. These results suggest that the mechanism responsible for AS may differ between Chinese and European patients, and that AS in Chinese patients may develop through a mechanism independent of these two IL-23R gene variants. Our results support the idea that different disease mechanisms exist in different ethnic populations [16], and a different predisposition gene may be involved in the IL-23/IL-23R signaling pathway in Chinese AS patients. However, we only investigated two SNPs in our study, and therefore cannot completely exclude a role for IL-23R gene in the development of AS in Chinese populations. Further studies including other SNPs that cover the IL-23R gene are necessary to clarify its true role in AS susceptibility.

## Conclusions

In conclusion, the results of this study differed substantially from those of previous European studies. We suggest that this discrepancy can be attributed to racial differences, indicating the need to perform genetic association studies in different ethnic populations to identify race-specific predisposition genes. However, the results of genetic association studies may also differ within races and may produce false-negative or false-positive results as a result of selection bias of the samples. Thus further studies in the Chinese Han population, including a larger sample size, are also required to confirm the effects of the SNP rs1004819 and SNP rs10889677 on the development of AS.

## Competing interests

The authors declare that they have no competing interests.

## Authors’ contributions

BPQ and JJ drafted the manuscript. MLJ carried out the genetic association study and the statistical analysis. BW and YY recruited the patients and participated in the design of this study. BPQ and YQ supervised this study and critically revised the manuscript. All the authors have read and approved the final manuscript.

## Pre-publication history

The pre-publication history for this paper can be accessed here:

http://www.biomedcentral.com/1471-2474/14/190/prepub
